# Extracorporeal liver assist device to exchange albumin and remove endotoxin in acute liver failure: Results of a pivotal pre-clinical study

**DOI:** 10.1016/j.jhep.2015.04.020

**Published:** 2015-09

**Authors:** Karla C.L. Lee, Luisa A. Baker, Giacomo Stanzani, Hatim Alibhai, Yu Mei Chang, Carolina Jimenez Palacios, Pamela J. Leckie, Paola Giordano, Simon L. Priestnall, Daniel J. Antoine, Rosalind E. Jenkins, Christopher E. Goldring, B. Kevin Park, Fausto Andreola, Banwari Agarwal, Rajeshwar P. Mookerjee, Nathan A. Davies, Rajiv Jalan

**Affiliations:** 1 Department of Clinical Science and Services, The Royal Veterinary College, Hertfordshire, UK; 2 Department of Pathology and Pathogen Biology, The Royal Veterinary College, Hertfordshire, UK; 3 Institute of Liver and Digestive Health, Liver Failure Group, University College London Medical School Royal Free Campus, London, UK; 4 MRC Centre for Drug Safety Science, Department of Molecular & Clinical Pharmacology, University of Liverpool, Liverpool, UK; 5 Intensive Care Unit, Royal Free London NHS Foundation Trust, London, UK

**Keywords:** LT, liver transplantation, ALF, acute liver failure, APAP, acetaminophen, NAPQI, N-acetyl-p-benzoquinone imine, DAMP, damage-associated molecular pattern, HMGB1, high-mobility group box-1 protein, DNA, deoxyribonucleic acid, TLR4, toll-like receptor 4, Nalp3, nacht, leucine-rich repeat and pyrin domain-containing protein 3, IL, interleukin, HAS, human serum albumin, MARS, Molecular Adsorbent Recirculating System, ACLF, acute-on chronic liver failure, UCL-LDD, University College London-Liver Dialysis Device, PALF, porcine model of acute liver failure, ICP, intracranial pressure, CVP, central venous pressure, INR, international normalised ratio, CD, Control Device, P_a_O_2_, partial pressure of oxygen in arterial blood, APAP-UCL-LDD, group treated with APAP and UCL-LDD, APAP-CD, group treated with APAP and CD, Control-CD, group treated with placebo, water and CD, HMA, non-oxidised human mercaptalbumin, HNA-1, reversibly oxidised human non-mercaptalbumin-1, HNA-2, irreversibly oxidised human non-mercaptalbumin-2, ELISA, enzyme-linked immunosorbent assay, AST, aspartate amino transferase, ALP, alkaline phosphatase, SVRI, systemic vascular resistance index, CI, cardiac index, SVI, stroke volume index, LVSWI, left ventricular stroke work index, RVSWI, right ventricular stroke work index, PCWP, pulmonary capillary wedge pressure, P_a_CO_2_, partial pressure of carbon dioxide in arterial blood, RR, respiratory rate, P_insp_, inspiratory pressures, SIRS, systemic inflammatory response syndrome, IL-1ra, IL-1 receptor antagonist, MAP, mean arterial pressure, HR, heart rate, P_a_O_2_/FiO_2_, ratio of partial pressure of oxygen in arterial blood to percentage of oxygen in inspired gases, PEEP, positive end expiratory pressure, Acute liver failure, Acetaminophen, Extracorporeal liver assist device, UCL-LDD, Albumin, Endotoxin, Toll-like receptor 4

## Abstract

**Background & Aims:**

In acute liver failure, severity of liver injury and clinical progression of disease are in part consequent upon activation of the innate immune system. Endotoxaemia contributes to innate immune system activation and the detoxifying function of albumin, critical to recovery from liver injury, is irreversibly destroyed in acute liver failure. University College London-Liver Dialysis Device is a novel artificial extracorporeal liver assist device, which is used with albumin infusion, to achieve removal and replacement of dysfunctional albumin and reduction in endotoxaemia. We aimed to test the effect of this device on survival in a pig model of acetaminophen-induced acute liver failure.

**Methods:**

Pigs were randomised to three groups: Acetaminophen plus University College London-Liver Dialysis Device (n = 9); Acetaminophen plus Control Device (n = 7); and Control plus Control Device (n = 4). Device treatment was initiated two h after onset of irreversible acute liver failure.

**Results:**

The Liver Dialysis Device resulted in 67% reduced risk of death in acetaminophen-induced acute liver failure compared to Control Device (hazard ratio = 0.33, *p* = 0.0439). This was associated with 27% decrease in circulating irreversibly oxidised human non-mercaptalbumin-2 throughout treatment (*p* = 0.046); 54% reduction in overall severity of endotoxaemia (*p* = 0.024); delay in development of vasoplegia and acute lung injury; and delay in systemic activation of the TLR4 signalling pathway. Liver Dialysis Device-associated adverse clinical effects were not seen.

**Conclusions:**

The survival benefit and lack of adverse effects would support clinical trials of University College London-Liver Dialysis Device in acute liver failure patients.

## Introduction

Liver transplantation (LT) is the only treatment proven to prolong survival in patients with acute liver failure (ALF), who fulfil criteria for poor prognosis, but LT remains a limited resource with alternative therapies being an unmet need [1]. In ALF, severity of liver injury and clinical progression of disease is in part consequent upon activation of the innate immune system [2], [3], [4], [5]. Endotoxaemia in ALF contributes to this innate immune response [6]. Moreover in liver failure, the detoxifying function of albumin, which is critical to recovery from liver injury, is irreversibly destroyed [7], [8].

Acetaminophen (APAP) overdose is the leading cause of ALF in the UK and USA [1], [9]. APAP toxicity results from its hepatic metabolism into toxic N-acetyl-p-benzoquinone imine (NAPQI). Subsequent formation of harmful NAPQI protein adducts causes mitochondrial oxidant stress and ultimately hepatocyte death [4]. However the severity of the ensuing liver injury and clinical syndrome of ALF is exacerbated by activation of the innate immune system by damage-associated molecular patterns (DAMPs) released by dying hepatocytes and likely other resident cells of the liver [3], [5]. DAMPs implicated in ALF include high-mobility group box-1 protein (HMGB1), heat shock protein 70 and deoxyribonucleic acid (DNA) fragments [10], [11], [12]. HMGB1 has been shown to act via the toll-like receptor 4 (TLR4) pathway to exacerbate immune response [12], [13], [14] and DNA fragments may contribute to activation of the nacht, leucine-rich repeat and pyrin domain-containing protein 3 (Nalp3) inflammasome and subsequent activation of pro-inflammatory cytokines, Interleukin-1β (IL-1β) and IL-18 [11].

Endotoxin or lipopolysaccharide, a component of the cell wall of gram-negative bacteria, has been described as a “cofactor” in APAP-induced liver injury [6]. The liver plays a critical role in clearance of gut-derived endotoxin, which may or may not be associated with viable gram-negative bacteria. Data from APAP-induced rodent models of ALF suggest that endotoxin stimulates the innate immune response and produces liver injury through activation of hepatic Kupffer cells to increase pro-inflammatory cytokine production [14], [15].

Albumin is the most abundant plasma protein and is synthesized in the liver. Besides maintaining plasma oncotic pressure, it also serves many detoxification, immune and circulatory functions [16]. Human serum albumin (HSA) infusion has been shown to reduce mortality in liver failure patients with spontaneous bacterial peritonitis and hepatorenal syndrome and following large volume paracentesis [16]. Molecular Adsorbent Recirculating System (MARS) is an extracorporeal liver assist device, based on the principle of albumin dialysis, which has been trialled extensively in patients with ALF and acute-on chronic liver failure (ACLF) but failed to show a survival benefit [17], [18], [19]. Prometheus, which is based on a similar principle, has been largely trialled in ACLF. Again, no survival benefit of this device could be demonstrated [18], [20]. University College London-Liver Dialysis Device (UCL-LDD) was developed based on the hypothesis that the ‘failure’ of MARS, Prometheus and other such devices may be partly due to their inability to reduce activation of the innate immune system [21], [22] and their inability to restore the function of circulating albumin molecules retained within the patient [7], [8], [23].

UCL-LDD is a novel artificial extracorporeal liver assist device, which includes a high cut-off filter for extraction of albumin, along with bound toxins, by haemofiltration and a selective endotoxin adsorption cartridge for selective endotoxin extraction by haemoperfusion. We hypothesised that UCL-LDD in combination with HSA infusion would prolong survival in ALF, not only by removal of water soluble and protein bound toxins, but also by selective reduction in endotoxemia, improvement in albumin detoxifying function and reduction in the innate immune response. Our aim was to interrogate our hypothesis by testing the effects of UCL-LDD compared to a Control Device delivering standard continuous renal replacement therapy in an APAP-induced porcine model of ALF.

## Material and methods

### Porcine model of acute liver failure (PALF)

A previously described pig model of APAP-induced ALF (PALF) was used in this study and has been described elsewhere [24]. Female, 26–36 kg, Landrace cross Large White pigs were used and all animal procedures complied with the animals (Scientific Procedures) Act 1986. Briefly, pigs were maintained under general anaesthesia with intermittent positive pressure ventilation. Pigs were instrumented for intravenous fluid and drug administration; arterial and venous blood sampling; and continuous monitoring of intracranial pressure (ICP), central venous pressure (CVP), direct arterial blood pressure, cardiac output and urine output. Critical care protocols for intravenous fluid therapy and maintenance of acid-base status, electrolyte balance, normoglycaemia, cardiovascular function and respiratory function were adhered to in order to maintain pre-defined physiological targets, detailed in Supplementary material [24]. However, albumin infusion was used according to a fixed protocol (see below).

Acute liver failure was induced in ‘APAP pigs’ with an aqueous APAP suspension, given via an oroduodenal tube. A loading dose of 0.25 g/kg APAP was followed by an hourly maintenance APAP dose, adjusted between 0.5 and 4 g to achieve toxic serum APAP concentrations of greater than 300 mg/L. APAP dosing was discontinued once the International Normalised Ratio (INR) exceeded three and this time point is hereafter referred to as ‘ALF’. This value was chosen as, in the PALF model, an INR of three is associated with 100% mortality. Thereafter, APAP pigs were treated with UCL-LDD or a ‘Control Device’ (CD) until non-recoverable cardiorespiratory arrest or 20 h after ALF. At 20 h after ALF, surviving animals were terminated with sodium pentobarbitone. ‘Control pigs’ were administered water without APAP for 20 h and then supported for a further 20 h prior to termination with sodium pentobarbitone. The 20 h end point was a requirement of the local ethics and welfare committee.

For survival studies, “death” was defined as time of non-recoverable cardiorespiratory arrest or when at least two of the following criteria were met: haematocrit <10%; blood potassium >5.5 mmol/L; blood lactate >10 mmol/L; blood pH <7.25; partial pressure of oxygen in arterial blood (P_a_O_2_) <60 mmHg. These alternative parameters for “death” were based on pilot study data, which showed that cardiorespiratory arrest always occurred within 1 to 3 h of occurrence of two of these parameters and that use of these terminal phase criteria to define death reduced variability in survival times and therefore number of pigs required for survival studies.

### UCL-LDD

All equipment and consumables were obtained from Gambro Dialysatoren GmbH, Rostock, Germany and used following the recommendations of this company. UCL-LDD incorporated two haemofilters: ‘SepteX’, a high cut-off filter for extraction of albumin, along with bound toxins, by haemofiltration and ‘OXiris’, a selective endotoxin adsorption cartridge for selective endotoxin extraction by haemoperfusion. Details of the UCL-LDD device are described in Supplementary material. UCL-LDD was compared to CD in which the two haemofilters of the UCL-LDD were replaced with standard continuous renal replacement haemofilters.

### Albumin infusion

In early pilot experiments, a marked reduction in serum albumin concentrations, to values less than 10 g/L, were detected in pigs treated with APAP, likely due to excessive capillary leak in this species [24]. Hence a fixed protocol for albumin infusion was developed as part of the PALF model to compensate for this. At onset of APAP dosing, intravenous infusion of 20% HSA (Bio Products Laboratory Ltd, Hertfordshire, UK) was initiated at 1.6 g albumin/h, this was increased 12 h later to 16 g albumin/h and further increased at ALF to 20 g albumin/h. Control pigs were given 1.6 g albumin/h from onset of water (placebo) administration. In both APAP and Control pigs, two units of porcine fresh frozen plasma were given at ALF or equivalent in order to prevent bleeding. After onset of device treatment in APAP pigs albumin infusion was discontinued if serum albumin exceeded 20 g/L. This albumin infusion protocol ensured appropriate (targeted to serum albumin concentrations) albumin replacement with UCL-LDD treatment.

### Study plan

Two studies, a pilot and main study, were performed sequentially.

### Pilot study. Efficacy of endotoxin and albumin removal by UCL-LDD

The pilot study determined whether the two filters could be used together and also to assess whether the individual filters were able to successfully (1) remove endotoxin resulting in a lowering of plasma endotoxin concentrations in the animals and (2) filter albumin and achieve an improvement in albumin function. The data for this pilot experiment is described in Supplementary material.

### Main study. Effect of UCL-LDD treatment on progression of ALF

To determine the effect of UCL-LDD treatment on survival, clinical progression, innate immune response and hepatocyte necrosis in APAP pigs following onset of ALF, 22 pigs were randomly allocated to one of three treatment groups: 1) nine pigs to the “APAP-UCL-LDD” group, induced to ALF with APAP and treated with UCL-LDD; 2) nine pigs to the “APAP-CD” group, induced to ALF with APAP and treated with CD; and 3) four pigs to the “Control-CD” group, sham induction to ALF with water and treated with CD. Number of pigs used was determined by power analysis to detect a 30% or greater increase in survival in APAP pigs with UCL-LDD compared to CD (5% type I error rate, 80% power). Extracorporeal device treatment was initiated 2 h after ALF. All researchers except one were blinded to the device being used. The researcher with knowledge of the device set up the device, concealing the cartridges and managed the study from the point of ALF to initiation of device treatment. All subsequent care of the model was carried out by blinded researchers. Clinical, biochemical and histopathological progression of disease was monitored as described previously [24]. Plasma (heparin) samples were collected before onset of APAP dosing, at ALF and every four hours thereafter and stored at −70 °C pending analysis.

### Kinetic turbidimetric limulus amebocyte lysate assay for endotoxin

Plasma endotoxin concentrations were measured using the Kinetic Turbidimetric Limulus Amebocyte Lysate Assay (Charles River Laboratories International Inc., MA, USA) according to the manufacturer’s instructions and as described previously [25].

### High performance liquid chromatography for HMA, HNA-1 and HNA-2

High performance liquid chromatography (JASCO UK Ltd, Essex, UK), using the Shodek Asahipak ES-502N 7C ion exchange column (Thames Restek UK Ltd, Buckinghamshire, UK), was used to determine the percentage of HSA present as the non-oxidised human mercaptalbumin (HMA) capable of free radical scavenging and molecule binding; the reversibly oxidised human non-mercaptalbumin-1 (HNA-1) and the irreversibly oxidised human non-mercaptalbumin-2 (HNA-2) in plasma samples, as previously described [8]. Albumin fractions were expressed as a percentage of the area under each peak relative to the total area under all three peaks using EZChrom Elite software (Agilent Technologies UK Ltd, Berkshire, UK).

### TLR4 and IL-18/IL-1β reporter cell assays

Systemic activation of the TLR4 signalling pathway and presence of bioactive IL-18 and IL-1β in plasma samples was assessed using ‘HEK-Blue hTLR4’ cells and ‘HEK-Blue IL-18/IL-1 β’ cells (InvivoGen, California, USA), respectively, according to the manufacturer’s instructions, as described previously [26] and detailed in Supplementary material.

### Total HMGB1 ELISA

Total HMGB1 in plasma samples was quantified by enzyme-linked immunosorbent assay (ELISA, Shino-Test Corporation, Tokyo, Japan) as previously described [10].

### Statistical analysis

Recordings of anaesthetic monitor data and drug and fluid infusion rates were recorded every 15 min, arterial blood gas data every hour, INR every one to four hours and biochemical analyses every four hours. Hourly to four hourly group summaries for each group (APAP-UCL-LDD, APAP-CD and Control-CD) were expressed as mean ± standard error and assessed graphically. To compare progression to ALF between the two APAP groups and the Control-CD group, linear mixed effects models and the two sample t-test were used. To assess the effect of UCL-LDD, group comparisons between APAP-UCL-LDD and APAP-CD were made for the first 8 h of treatment i.e. ALF + 2 h to ALF + 10 h. First degree auto-regressive or compound symmetry (co)variance structure was used to account for the correlation between hourly or 4-hourly repeated measures. These analyses were carried out using the generalised linear and mixed model procedures in SAS version 9.1 for PC (Copyright, SAS Institute Inc. Cary, NC, USA) and significance was set at the 5% level.

Assay results for endotoxin, redox states of albumin, systemic TLR4 activity, bioactive IL-18/IL-1 β and HMGB1 were available up to every 4 h from point of ALF. For endotoxin, redox states of albumin and HMGB1 assays, it was not possible to run samples from all animals in each group: for these assays, animals included were based on a randomised block design, dependent on the three study groups and number of samples which could be included in each assay plate. To assess the effect of APAP and treatment, group comparisons between APAP-UCL-LDD, APAP-CD and Control-CD were made. First degree auto-regressive or compound symmetry (co)variance structure was used to account for the correlation between repeated measures. Analyses were carried out using the generalised linear and mixed model procedures in SAS version 9.1 for PC (Copyright, SAS Institute Inc. Cary, NC, USA) and significance was set at the 5% level.

To compare survival time between APAP-UCL-LDD and APAP-CD, Cox regression and Kaplan-Meier survival curve analyses were performed using R 3.0.0 (Institute for Statistics and Mathematics, Wirtschaftsuniversität, Vienna, Austria).

## Results

### Survival

All nine APAP-UCL-LDD pigs, seven out of nine APAP-CD pigs and all four Control-CD pigs completed the study. Two APAP-CD pigs were excluded due to early achievement of ALF criteria at 12.5 and 13 h after onset of APAP dosing, more than 30% below the mean of 19.3 ± 1.8 expected for the PALF model. In the nine APAP-UCL-LDD pigs and seven APAP-CD pigs that completed the study mean serum APAP concentrations exceeded 300 mg/L within 4 h of onset of APAP dosing and INR >3, ALF, was recorded at 18.4 ± 0.6 h. Total dose of acetaminophen administered to achieve ALF was 50.7 ± 3.0 g and 49.2 ± 4.0 g in the APAP-CD and APAP-UCL-LDD groups respectively.

**Fig. 1:**
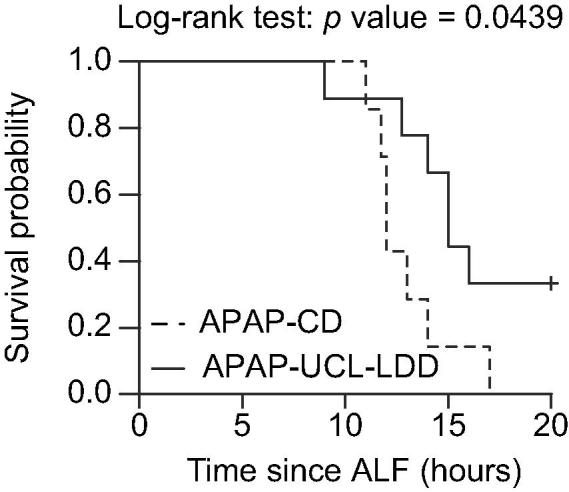
**UCL-LDD reduces risk of death in ALF.** Effect of UCL-LDD (solid black line) on survival compared to CD (broken black line).

All APAP-CD pigs progressed to “death” within the 20 h study period from ALF, with a median survival time from ALF of 12 h (range 11–17 h). In the APAP-CD group “death” was achieved in five by cardiorespiratory arrest and in two by achievement of two “death” criteria: blood pH <7.25 with haematocrit <10% in one pig and blood pH <7.25 with lactate >10 mmol/L and haematocrit <10% in one pig. Three out of nine APAP-UCL-LDD pigs “survived” to the end of the study, resulting in an overall estimated median survival time from ALF for APAP-UCL-LDD pigs of 15 h (range 9–20 h). In the APAP-UCL-LDD group, “death” was achieved in one by cardiorespiratory arrest and in five by achievement of two “death” criteria: blood pH <7.25 with one or more of haematocrit <10%, blood potassium >5.5 mmol/L and blood lactate >10 mmol/L. Therefore UCL-LDD resulted in a 67% reduced risk of “death” compared to CD (hazard ratio = 0.33, *p* = 0.0439, Fig. 1).

### Liver, cerebral and renal function

Acute centrilobular to midzonal hepatocyte degeneration and necrosis was confirmed at post mortem examination in all APAP pigs. All Control-CD pigs had normal livers. Necrosis was graded as mild, moderate or severe according to percentage of parenchyma affected: there was no significant difference in severity of necrosis between UCL-LDD and CD (Supplementary material 5). Elevation in liver enzymes, aspartate amino transferase (AST) and alkaline phosphatase (ALP), was not seen in this animal model of ALF as reported previously (Table 1) [24].

**Table 1:** **Kidney and liver biochemistry profiles.**

Kidney and liver biochemistry profiles in APAP-UCL-LDD, APAP-CD and Control-CD groups at ALF, every 4 h thereafter and at the 4 h time point directly preceding death. (‘-’ denotes insufficient sample number for comparison due to death).

Intracranial pressure increased significantly from onset of ALF to death from 17 ± 1 to 32 ± 1 mmHg (*p* <0.0001) in the APAP-UCL-LDD group and from 16 ± 2 to 33 ± 3 mmHg (*p* <0.0001) in the APAP-CD group. This change was not significantly different between groups.

Serum creatinine was significantly higher in the APAP groups compared to the Control-CD group at ALF (Table 1, *p* = 0.0004). During the following 10 h there was a mild decrease in creatinine concentrations in both APAP-UCL-LDD and APAP-CD groups (*p* = 0.0237), but with no significant difference between devices. Post mortem kidney examination revealed varying degrees of tubular epithelial cell swelling and protein loss in both the APAP-UCL-LDD and APAP-CD groups, but no renal pathology in the Control-CD group.

### Cardiovascular function

**Fig. 2:**
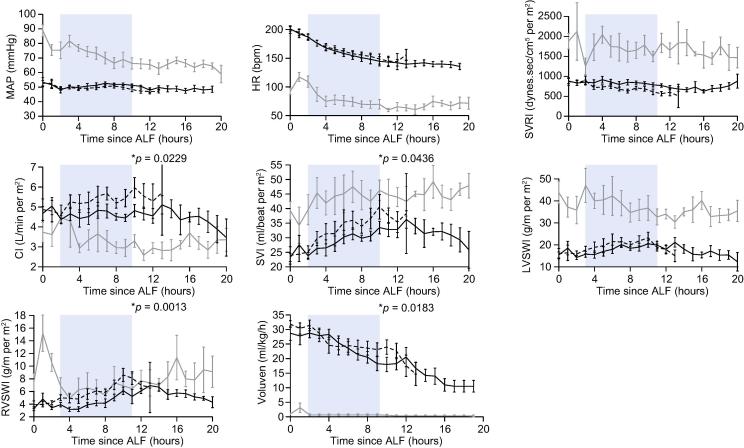
**UCL-LDD improves cardiovascular function in ALF.** UCL-LDD delayed vasoplegia/high output cardiac failure and reduced severity of cardiovascular failure during the first 8 h of treatment i.e. 2–10 h after ALF (blue shading) in the APAP-UCL-LDD group (solid black line) compared to APAP-CD Group (broken black line) [Control-CD Group (grey line)]. (ALF, time INR exceeded 3; MAP, mean arterial pressure; HR, heart rate; SVRI, systemic vascular resistance index; CI, cardiac index; SVI, stroke volume index; LVSWI, left ventricular stroke work index; RVSWI, right ventricular stroke work index; Voluven, colloid therapy; ^∗^, significant (*p* <0.0500) difference between APAP-UCL-LDD and APAP-CD).

During the first 8 h of device treatment, both APAP groups demonstrated: peripheral vasodilation (hypotension, *p* <0.0001) with low systemic vascular resistance index (SVRI, *p* <0.0001); tachycardia (*p* <0.0001); a high cardiac output failure state [increased cardiac index (CI, *p* = 0.0069), stroke volume index (SVI, *p* <0.0001), left ventricular stroke work index (LVSWI, *p* = 0.0164) and right ventricular stroke work index (RVSWI, *p* <0.0001)]; and elevation in CVP (*p* <0.0001) and pulmonary capillary wedge pressure (PCWP, *p* = 0.0011) associated with development of peripheral oedema. In the APAP-UCL-LDD group, development of systemic vasoplegia, high cardiac output and hyperdynamic circulatory state was less severe and colloid requirements were lower compared to the APAP-CD group. These differences were significant after 8 h of device treatment (Fig. 2).

### Respiratory function

**Fig. 3:**
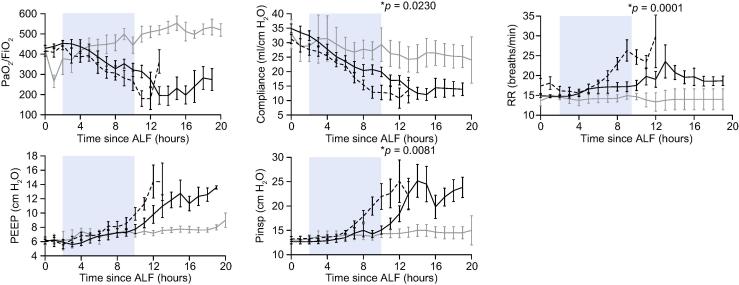
**UCL-LDD delays onset of acute lung injury in ALF.** UCL-LDD resulted in a delay in respiratory failure during the first 8 h of treatment i.e. 2–10 h after ALF (blue shading) in the APAP-UCL-LDD group (solid black line) compared to APAP-CD Group (broken black line) [Control-CD Group (grey line)]. (ALF, time INR exceeded 3; P_a_O_2_/FiO_2_, ratio of partial pressure of oxygen in arterial blood to percentage of oxygen in inspired gases; Compl, lung compliance; RR, respiratory rate; PEEP, positive end expiratory pressure; P_insp_, inspiratory pressure; ^*^, significant (*p* <0.0500) difference between APAP-UCL-LDD and APAP-CD).

During the first 8 h of device treatment, both APAP groups demonstrated an increasing requirement for mechanical ventilator support to achieve control of minute ventilation, P_a_CO_2_ and oxygenation (Fig. 3). This increasing requirement was delayed in the APAP-UCL-LDD group compared to the APAP-CD group, resulting in significantly higher lung compliance (Compliance), lower respiratory rate (RR) and lower inspiratory pressures (P_insp_) 8 h after onset of device treatment (Fig. 3).

### Endotoxin removal and albumin exchange

**Fig. 4:**
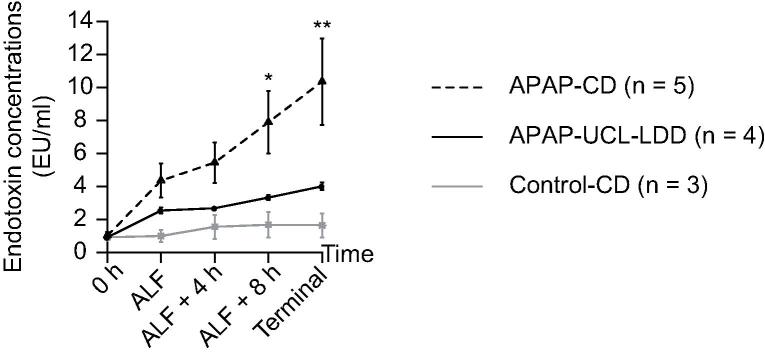
**UCL-LDD reduces endotoxaemia in ALF.** Endotoxin concentrations measured in plasma samples from APAP-CD, APAP-UCL-LDD and Control-CD pigs at time zero (0 h), ALF, ALF + 4 h, ALF + 8 h and the last blood sampling point prior to death (‘Terminal’). All plasma samples were diluted 1 in 10 and spiked with 5EU endotoxin. Endotoxin concentrations at ALF + 8 h (^*^p = 0.018) and ‘Terminal’ (^**^p = 0.002) were significantly lower in the APAP-UCL-LDD group compared to the APAP-CD group.

Endotoxin removal by UCL-LDD resulted in a reduction in increase in plasma endotoxin concentrations from ALF to death in the APAP-UCL-LDD group (57.3 ± 15.2% increase) compared to the APAP-CD group (119.3 ± 13.8% increase) (*p* = 0.024) (Fig. 4).

Serum albumin concentration was significantly lower in both APAP groups compared to Control-CD (*p* <0.0001) at ALF and during the following 12 h. However there was no significant difference in serum albumin concentrations between APAP-UCL-LDD and APAP-CD groups during this time period (Table 1). Total albumin dose administered up to ALF was 126 ± 14 g, 129 ± 8 g and 32 ± 0 g in APAP-UCL-LDD, APAP-CD and Control-CD groups respectively. Total albumin dose administered during the first 12 h after ALF, which included the first 10 h of device treatment, was 240 g for both APAP-UCL-LDD and APAP-CD groups and 19.2 g for the Control-CD group. Six and 10 h after onset of device treatment, percentage of serum albumin present in the HNA-2 form was significantly lower in the APAP-UCL-LDD group compared to the APAP-CD group (*p* = 0.023 and *p* = 0.046 respectively, Table 2).

**Table 2:** **Change in percentage of human albumin present as HMA, HNA-1 and HNA-2 with time at ALF and times after onset of device treatment in the APAP-UCL-LDD and APAP-CD groups.**

Device treatment was started 2 h after onset of ALF. *p* values are for comparisons between APAP-UCL-LDD and APAP-CD and are given where *p* <0.050.

### Activation of the TLR4 signalling pathway and IL-18/IL-1β bioactivity

**Fig. 5:**
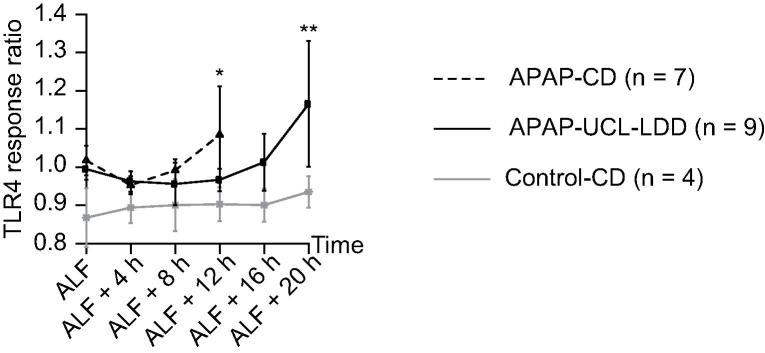
**UCL-LDD delays activation of the TLR-4 signalling pathway in ALF.** Response ratios (relative to negative control) for TLR4 reporter cell assay. Response ratio for positive control was 3.196 ± 0.369. Response ratio for APAP-CD was only significantly increased compared to Control-CD at ALF + 12 h (^*^p = 0.040). Response ratio for APAP-UCL-LDD was only significantly increased compared to Control-CD at ALF + 20 h (^**^p = 0.030), representing a delay in TLR4 activation by UCL-LDD treatment.

Activation of TLR4 signalling in the reporter cell lines was observed with plasma from both APAP groups. In the APAP-CD group this activation became significant at 12 h after onset of ALF (*p* = 0.040, Fig. 5). However in the APAP-UCL-LDD group, UCL-LDD treatment delayed activation of the TLR4 signalling pathway to 20 h after onset of ALF i.e. 18 h after onset of UCL-LDD treatment (*p* = 0.030, Fig. 5).

No significant IL-18/IL-1β bioactivity was detected in any of the plasma samples obtained after onset of ALF from animals in the APAP-CD and APAP-UCL-LDD groups when compared to animals in the Control-CD group (Supplementary material).

### Total HMGB1 plasma concentrations

**Fig. 6:**
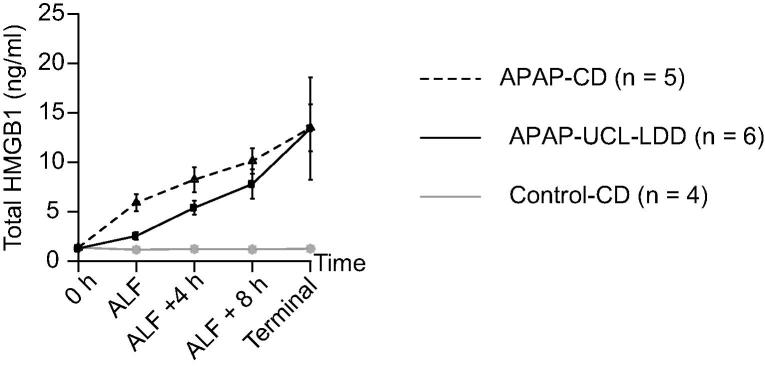
**UCL-LDD has no effect on total HMGB1 plasma concentrations in ALF.** Total HMGB1 concentrations measured in plasma samples from APAP-CD, APAP-UCL-LDD and Control-CD pigs at 0 h, ALF, ALF + 4 h, ALF + 8 h and the last blood sampling point prior to death (‘Terminal’). Data was log transformed for statistical analysis. There was a significant increase in total HMGB1 in APAP-CD and APAP-UCL-LDD groups with time (*p* <0.001) but not in the Control-CD group. After onset of ALF, UCL-LDD treatment did not significantly affect further increase in HMGB1 compared to CD treatment.

There was no significant change in total HMGB1 plasma concentration in the Control-CD group with time. In the APAP-CD and APAP-UCL-LDD groups, total HMGB1 plasma concentration increased significantly with progression of ALF to death (*p* <0.001) with no significant differences between groups (Fig. 6).

## Discussion

This study shows that UCL-LDD, an extracorporeal liver assist device, combined with HSA infusion is able to attenuate increase in circulating endotoxin concentrations, effect albumin exchange and decrease percentage of circulating HNA-2 in PALF. By so doing, UCL-LDD prolongs survival in ALF, associated with attenuation in the development of circulatory failure and acute lung injury. This clinical improvement is associated with a delay in systemic activation of the TLR4 signalling pathway. Adverse clinical effects were not seen compared to standard continuous renal replacement therapy. The survival benefit and lack of adverse effects would support clinical trials of UCL-LDD in ALF patients.

Artificial extracorporeal liver support devices, with the primary aim of replacing the detoxifying functions of the liver, have been in development since the 1950’s [27], but an improvement in survival of liver failure patients with these devices has remained elusive [17], [19], [20], [28]. The specific design criteria for UCL-LDD compared to previous liver dialysis devices were not only to achieve non-selective removal of water soluble and protein bound toxins, but also selective reduction in endotoxaemia and albumin replacement, in order to improve albumin detoxifying function and reduce the innate immune response. These criteria were based on a number of observations in liver failure patients: 1) irreversible loss of the detoxifying function of albumin in ACLF [7], [8]; 2) significant association between loss of albumin function and mortality risk in ACLF [7], [8]; 3) significant association between infection/endotoxaemia and morbidity and mortality in ACLF and ALF [29], [30], [31]; and 4) increasing evidence to support the role of endotoxin in the innate immune response in ALF, which worsens liver injury and clinical liver failure [14], [15], [26]. In keeping with the hypothesis, treatment was associated with a survival advantage in ALF pigs. However, due to the pre-determined termination of all studies 20 h after ALF, it is not possible to say whether UCL-LDD treatment may have permitted recovery in those animals that ‘survived’. From the clinical perspective this survival advantage was due to the effect of the device on cardiac and pulmonary function rather than improvement in intracranial hypertension or liver necrosis. Failure to demonstrate improvement in liver necrosis is not surprising as treatment with UCL-LDD was initiated when ALF was fully established.

The potential role of various continuous renal replacement therapy techniques to reduce the innate immune response in critically ill patients has been widely studied in sepsis and systemic inflammatory response syndrome (SIRS) [32]. Indeed both of the haemofilters incorporated within UCL-LDD have been investigated in sepsis and SIRS.

In this study, the filter, SepteX, employed to achieve removal of albumin and albumin bound toxins, has a nominal cut-off of 60 kDa. However, *in vitro* and *in vivo* studies have demonstrated albumin (64 kDa) losses with this filter [33], [34]. The albumin loss of 2.4 g/h in the current study is in line with these previous reports. However SepteX was actually designed for removal of pro and anti-inflammatory cytokines up to 45 kDa in states of excessive inflammation. In septic patients with acute renal failure, SepteX has been shown to decrease plasma concentrations of IL-6 and IL-1 receptor antagonist (IL-1ra), correct immune dysfunction and reduce the need for vasopressor support compared to standard renal replacement therapies [35], [36], [37]. Therefore the beneficial effect of SepteX in UCL-LDD may be due to removal of dysfunctional albumin, but also dampening of the innate immune response implicated in ALF.

UCL-LDD treatment plus HSA infusion was associated with a significant decrease in the irreversibly destroyed form of albumin, the HNA-2 form. The decrease reported was of the order of magnitude previously demonstrated to be prognostic for patients with advanced liver disease [8]. It is likely that the reduction in HNA-2 resulted from removal of HNA-2 by SepteX and replacement with non-oxidised albumin. In addition, improvement in systemic oxidative stress by UCL-LDD may have resulted in decreased oxidative albumin damage. Improvement in albumin function, clearance of albumin bound toxins and consequent reduction in toxin-associated tissue injury likely contributed to improved survival.

The selective endotoxin adsorption filter, OXiris was used with SepteX in UCL-LDD, as our preliminary data clearly demonstrate that OXiris alone inhibited progression of endotoxaemia in ALF, but that SepteX alone did not. Oxiris has been shown previously in a pig model of sepsis to decrease plasma IL-6 concentrations and improve cardiovascular function [38] in agreement with the data presented in the current study.

Cell response assays were used to assess innate immune cell activation in this study, as they may more accurately reflect the balance of pro and anti-inflammatory mediators within the plasma compared to the measurement of individual cytokine concentrations. Numerous studies have demonstrated that certain renal replacement therapies result in significant cytokine clearance, associated with clinical improvement in critical illness, without significant effect on circulating cytokine concentrations, which may be due to reductions in tissue level concentrations of pro and anti-inflammatory mediators and a return to near normal immune homeostasis at the tissue level [38]. In this study we document delay in systemic activation of the TLR4 signalling pathway in ALF by UCL-LDD treatment, which concurrently attenuates rise in endotoxaemia and improves clinical signs of systemic inflammation. This agrees with data from rodent models which suggest that endotoxin activates cellular receptors including TLR4 on hepatic Kupffer cells to exacerbate APAP-induced liver injury and clinical liver failure [14], [15], [26]. However the current study does not provide a direct assessment of innate immunity within the liver. Future studies to explore the mechanisms underlying the beneficial effect of UCL-LDD should focus on the liver, using techniques such as microdialysis [39].

The finding that CD and UCL-LDD treatment did not decrease total HMGB1 plasma concentrations was unexpected. Previous studies show that filtration with a high cut-off membrane, may not achieve HMGB1 clearance [40]. However AN69 ST, a membrane similar to OXiris, was able to achieve 100% HMGB1 clearance *in vitro* by adsorption [40]. HMGB1 exists in a number of isoforms with post-translational modifications governing subcellular localisation and release from cells and inflammatory function [41], [42], [43], [44]. It is possible, that in this study, improved survival was associated with selective removal of a specific functionally relevant HMGB1 isoform, which was not reflected in total HMGB1 plasma concentration. Mass spectrometry based profiling of HMGB1 isoforms would be useful to investigate this further.

## Conclusion

In a pig model of APAP-induced ALF, we have shown that UCL-LDD, a novel extracorporeal liver assist device, in combination with HSA infusion, results in a decrease in circulating irreversibly oxidised albumin (HNA2) and a reduction in the overall severity of endotoxaemia. This attenuated the severity of multi-organ dysfunction, resulting in prolonged survival and an estimated 67% reduced risk of death compared to controls, associated with a delay in systemic activation of the TLR4 signalling pathway. No adverse effect of the device or compatibility issues were observed providing rationale for clinical trials of this device.

## Financial support

This study was supported by the UK Medical Research Council Developmental Pathway Funding Scheme Award, G0902211. Equipment for continuous renal replacement therapy and the University College London-Liver Dialysis Device was provided by Gambro Dialysatoren GmbH, Rostock, Germany. Human serum albumin was provided by Bio Products Laboratory Ltd, Hertfordshire, UK.

## Conflict of interest

The authors have nothing to declare with the following exceptions. Rajiv Jalan and Nathan Davies are the inventors of University College London-Liver Dialysis Device, which has been patented by University College London and licensed to Yagrit Limited. Rajiv Jalan has research collaborations with Ocera, Grifols, Norgine and Gambro, consults for Ocera and Conatus and has received speaking fees from Norgine and Grifols.

## Author’s contributions

KCLL – overall study concept and design; execution and management of studies; data acquisition, analysis and interpretation; principle author of manuscript; obtained funding.
LAB – execution and management of studies; data acquisition; critical revision of manuscript.
GS – execution and management of studies; data acquisition; critical revision of manuscript.
HA – study concept and design relating to PALF; execution and management of studies; data acquisition; obtained funding.
YMC – statistical support and analysis; critical revision of manuscript.
CPJ – study concept and design relating to PALF.
PJL – study concept and design relating to University College London-Liver Dialysis Device; technical support.
PG – execution and management of studies; data acquisition.
SLP – histopathological analysis; data acquisition; critical revision of manuscript.
DJA – execution of HMGB1 assays; data acquisition; critical revision of manuscript.
REJ – execution of HMGB1 assays; data acquisition.
CEP – execution of HMGB1 assays; data acquisition.
BKP – execution of HMGB1 assays; data acquisition.
FA – study concept and design relating to reporter cell assays; execution and management of reporter cell assays; data acquisition.
BA – data interpretation; critical revision of manuscript.
RPM – overall study concept and design; data interpretation; critical revision of manuscript; obtained funding.
NAD – overall study concept and design; execution and management of studies; data interpretation; critical revision of manuscript; obtained funding.
RJ – overall study concept and design; data interpretation; critical revision of manuscript; obtained funding.
