# Supplementary material for: Extracorporeal liver assist device to exchange albumin and remove endotoxin in acute liver failure: Results of a pivotal pre-clinical study
Source: J Hepatol. 2015 Sep;63(3):634–42. doi: 10.1016/j.jhep.2015.04.020 (PMC4541472; doi:10.1016/j.jhep.2015.04.020)
Supplement: Supplementary data [file mmc1.pdf]

Supplementary data to:

Extracorporeal liver-assist device to exchange albumin and remove endotoxin in acute liver failure: Results of a pivotal pre-clinical study

Karla C L Lee[1,*], Luisa A Baker[1], Giacomo Stanzani[1], Hatim Alibhai[1], Yu Mei Chang[1], Carolina Jimenez Palacios[1], Pamela J Leckie[3], Paola Giordano[1], Simon L Priestnall[2], Daniel J Antoine[4], Rosalind E Jenkins[4], Christopher E. Goldring[4], B Kevin Park[4], Fausto Andreola[3], Banwari Agarwal[5], Rajeshwar P Mookerjee[3], Nathan A Davies[3], Rajiv Jalan[3]

Table of Contents

1. Critical care protocols used in the porcine acetaminophen-induced model of acute liver failure for: ventilation; intravenous fluid therapy; maintenance of acid-base status, electrolyte balance, and normoglycaemia; and cardiovascular support 3

2. University College London Liver Dialysis Device (UCL-LDD): technical details 5

3. Pilot study: efficacy of endotoxin and albumin removal by University College London-Liver Dialysis Device 6

4. Methods for reporter cell assays 9

TLR4 reporter cell assay 10

IL-18/IL-1β reporter cell assay 11

5. Liver histopathology results 11

6. Results for Il-18/Il-1β reporter cell assays 12
Figures 13
Fig. 1: Schematic representation of University College London-Liver Dialysis Device (UCL-LDD). 13
Fig. 2: Effect of treatment with four haemofilter combinations on endotoxin concentrations measured in plasma samples from pigs with acetaminophen-induced acute liver failure. 14
Fig. 3 Post mortem examination of liver specimens from pigs included in the main study to assess the efficacy of treatment with University College London-Liver Dialysis Device (UCL-LDD) compared to a Control Device (CD) in pigs with acetaminophen-induced acute liver failure.. 16
Fig. 4 IL-1b/IL-18 activity in plasma from acute liver failure pigs treated with either University College London-Liver Dialysis Device or Control Device 17
References.. 18

1. Critical care protocols used in the porcine acetaminophen-induced model of acute liver failure for: ventilation; intravenous fluid therapy; maintenance of acid-base status, electrolyte balance, and normoglycaemia; and cardiovascular support

Critical care management of the porcine acetaminophen-induced model of acute liver failure has been previously published [1]. The following is a summary of the critical care protocols for ventilation; intravenous fluid therapy; maintenance of acid-base status, electrolyte balance, and normoglycaemia; and cardiovascular support.

Ventilation

Pressure-controlled intermittent positive pressure ventilation with a circle breathing system and a mixture of oxygen and medical air was used throughout. Gas flow rate was maintained throughout at 20-22 ml/kg/min. At beginning of anaesthesia, ratio of oxygen to medical air was set to maintain percentage of inspired oxygen at 30-36%, positive end expiratory pressure was 5 cmH_2O, inspired pressure was 12-15 cmH_2O and respiratory rate was 10-13 breaths per minute. Three consecutive sighs were introduced manually every one to two hours. Ventilator controls were adjusted to maintain partial pressure of carbon dioxide in arterial blood between 35-45 mmHg, partial pressure of oxygen in arterial blood between 100-120 mmHg and tidal volume between 6-10 ml/kg.

Intravenous fluid therapy and correction of electrolyte and acid-base abnormalities

Intravenous fluid therapy with crystalloid solutions was administered at a constant rate of 5ml/kg/hr throughout. Crystalloid solutions used were compound sodium

lactate from induction of anaesthesia; 0.9% sodium chloride from 12 hours after onset of acetaminophen dosing; and 0.18% sodium chloride with 4% glucose if arterial blood sodium concentrations exceeded 145 mmol/l and continued to increase towards 150 mmol/l.

6% hydroxyethyl starch (‘Voluven 6%’, Fresenius Kabi Ltd, Cheshire, UK) was administered intravenously at 1 ml/kg/hr from onset of acetaminophen dosing. Rate was then adjusted to maintain mean arterial blood pressure as detailed below.

1 ml/kg/hr of a parenteral nutrition solution containing 10% glucose and 5.9% amino acids (‘Vamin 9 Glucose’, Fresenius Kabi Ltd, Cheshire, UK) was administered intravenously from onset of acetaminophen dosing until study end. Further intervention to maintain normoglycaemia was not required.

Potassium chloride was added to intravenous crystalloid solutions, as required, if arterial blood potassium concentrations fell below 3.5 mmol/l. Potassium infusion was stopped if arterial blood potassium exceeded 3.5 mmol/l and continued to increase towards 5 mmol/l.

8.4 % Sodium bicarbonate was given by intravenous infusion to correct metabolic acidosis.

10 % calcium gluconate was given slowly to effect by intravenous infusion if arterial blood calcium fell below 1.1 mmol/l.

Cardiovascular support

Direct mean arterial blood pressure (MAP) was monitored continuously with the aim to maintain MAP at >70 mmHg for the first 12 hours following onset of acetaminophen dosing, then >60 mmHg until onset of irreversible acute liver failure (ALF), then >50 mmHg until study end. If MAP fell below these targets three possible actions were taken. First, a 5ml/kg intravenous bolus of 6% hydroxyethyl starch was given over 15 minutes. This was repeated up to 3 times as long as central venous pressure (CVP) remained <12 mmHg. Following achievement of MAP targets, continuous rate infusion of 6% hydroxyethyl starch was increased by 1 ml/kg/hr. Second, intravenous noradrenaline infusion was initiated at 0.1 µg/kg/min and increased by 0.1 µg/kg/min every 15 minutes until MAP targets were achieved. Third, intravenous terlipressin infusion was initiated at 2 mg/24 hours when noradrenaline exceeded 0.5 µg/kg/min and further increased to 4 mg/24 hours when noradrenaline exceeded 1.0 µg/kg/min. If following irreversible ALF, MAP >50 mmHg was maintained for 1 h, reduction in 6% hydroxyethyl starch infusion was attempted if CVP was >9 mmHg. However if CVP was <9 mmHg, decrease in noradrenaline infusion was attempted. Both reductions were attempted whilst maintaining MAP >50 mmHg.

2. University College London-Liver Dialysis Device (UCL-LDD): technical details

UCL-LDD is a novel artificial extracorporeal liver support device including two haemofilters, Filter 1 and Filter 2. All components of the device were obtained from Gambro Dialysatoren GmbH, Rostock, Germany and used following the

recommendations of this company. A schematic of the device is shown in Fig. 1. Filter 1 was 'SepteX^{TM}', a haemofilter with a polyarylethersulfone 'high cut-off' membrane (effective surface area 1.1 m^2, pore diameter ~10 nm, membrane thickness 50 µm, internal diameter 215 µm). SepteX^{TM} was included to achieve extraction of albumin, along with bound toxins, by haemofiltration [2]. Filter 2 was 'OXiris^{TM}', a haemofilter containing an acrylonitrile and sodium methallyl sulfonate copolymer membrane, surface-treated with a cationic polymer, polyethylenimine for selective endotoxin removal by adsorption and heparin grafted (effective surface area 1.5 m^2, membrane thickness 50 µm, internal diameter 240 µm). OXiris^{TM} was included to achieve selective endotoxin extraction by haemoperfusion [Gambro Dialysatoren GmbH internal data].

UCL-LDD was compared to a 'Control Device (CD)' in which the two haemofilters of the UCL-LDD were replaced with 'HF1000^{TM}', a standard continuous renal replacement polyarylethersulphone hemofilter (effective surface area 1.1 m^2, membrane thickness 50 µm, internal diameter 215 µm).

Treatment with both UCL-LDD and CD was via a femoral haemodialysis catheter. 'PrimaSol BGK 2/0' was used for the replacement fluid and dialysate. Blood flow rate was set at 150ml/min, replacement (pre blood pump) volume at 1 l/h and dialysate rate at 35ml/kg/h, with zero net fluid removal. The circuit was anticoagulated continuously with 1000 iu heparin/h.

3. Pilot study: efficacy of endotoxin and albumin removal by University College London-Liver Dialysis Device

Aims

To determine the efficacy of albumin removal and endotoxin adsorption from pigs with acetaminophen (APAP)-induced acute liver failure (ALF) by the high cut-off filter, SepteX^{TM} (Gambro Dialysatoren GmbH, Rostock, Germany) and the endotoxin adsorption filter, OXiris^{TM} (Gambro Dialysatoren GmbH, Rostock, Germany) in University College London-Liver Dialysis Device (UCL-LDD).

Methods

The efficacy of albumin removal and endotoxin adsorption from pigs with APAP-induced ALF by the SepteX^{TM} and OXiris^{TM} haemofilters within UCL-LDD, was assessed by exchanging and thus comparing these filters with a standard continuous renal replacement filter, HF1000^{TM} (Gambro Dialysatoren GmbH, Rostock, Germany). Twelve pigs were induced to ALF with APAP and divided into four treatment groups of n=3. Each group underwent extracorporeal device treatment with a combination of two haemofilters beginning 2 h after onset of ALF.

- Group 1 was treated with SepteX^{TM} plus OXiris^{TM} that is UCL-LDD
- Group 2 was treated with SepteX^{TM} plus HF1000^{TM}
- Group 3 was treated with HF1000^{TM} plus OXiris^{TM}
- Group 4 was treated with HF1000^{TM} plus HF1000^{TM} that is the Control Device (CD)

Extracorporeal device treatment was via a femoral haemodialysis catheter. ‘PrimaSol BGK 2/0’ was used for the replacement fluid and dialysate. Blood flow rate was set at 150ml/min, replacement (pre blood pump) volume at 1 l/h and dialysate rate at 35ml/kg/h, with zero net fluid removal.

Plasma (heparin) samples were collected before onset of APAP dosing (baseline), at ALF and two and six h after onset of extracorporeal device treatment. Samples were stored at -70 °C pending analysis.

Plasma endotoxin concentrations were measured in samples diluted 1 in 10 with endotoxin free water and heat treated at 70 °C for 15 mins, to which a spike of 5EU endotoxin was added, using the Kinetic Turbidimetric Limulus Amebocyte Lysate Assay (Charles River Laboratories International Inc., MA, USA) according to the manufacturer's instructions. Plasma endotoxin concentrations were derived from standard curves using a polynomial regression model according to the assay manufacturer's recommendations.

Effluent samples were collected from three pigs treated with SepteX™ and three pigs treated without SepteX™, two and six h after onset of extracorporeal device treatment. Samples were stored at -70 °C pending analysis. Albumin concentrations were measured using a Cobas biochemistry analyser (Roche Diagnostics Ltd, Burgess Hill, UK).

Endotoxin and albumin concentrations were compared between treatment groups and over time using a linear mixed effects model. Analysis was carried out using IBM SPSS version 20 (New York, USA) and significance was set at the 5% level.

Results

Measured plasma endotoxin concentrations are illustrated in Fig. 2 for the four treatment groups. In all four groups there was a significant 362 ± 39 % increase in

plasma endotoxin from 2.6 ± 0.7 EU/ml at baseline to 11.6 ± 3.8 EU/ml at ALF ($p < 0.001$). In the CD and SepteX™ plus HF1000™ groups, endotoxin concentrations continued to rise significantly through the 6 h treatment period by 98 ± 50 % ($p < 0.001$) and 81 ± 7 % ($p = 0.004$) respectively. However in the UCL-LDD and the HF1000™ plus OXiris groups there was no significant increase in endotoxin concentrations after initiation of treatment.

Effluent albumin concentrations were significantly higher with SepteX™ than without SepteX™ ($p = 0.032$). Without SepteX™ effluent albumin concentrations at 2 and 6 h of treatment were 0.42 ± 0.21 g/l and 0.68 ± 0.14 g/l respectively. With SepteX™ effluent albumin concentrations at 2 and 6 h of treatment were 1.22 ± 0.10 g/l and 1.20 ± 0.15 g/l respectively. Effluent albumin concentrations were not significantly affected by time of treatment. With an effluent volume of 2 l/h, over a 6 h treatment period, total albumin extraction with SepteX™ was 2.42 ± 0.14 g/h.

Conclusion

In pigs with APAP-induced ALF, treatment with UCL-LDD achieves albumin extraction at a rate of 2.42 ± 0.14 g/h due to the presence of the SepteX™ filter and prevents further increase in plasma endotoxin concentrations due to the presence of the OXiris™ filter.

4. Methods for reporter cell assays

TLR4 reporter cell assay

Systemic activation of the TLR4 signalling pathway was assessed by exposure of HEK293 cells stably transfected with the hTLR4 gene, the MD-2/CD14 co-receptor genes and a secreted embryonic alkaline phosphatase (SEAP) reporter gene ('HEK-Blue hTLR4 Cells', InvivoGen, California, USA) to plasma samples from the experimental animals, according to the manufacturer's instructions. A cell suspension containing 100 000 cells/ml in DMEM containing 4.5 g/l glucose, 10 % (v/v) heat-inactivated fetal bovine serum, 50 U/ml penicillin, 50 µg/ml streptomycin, 100 µg/ml Normocin and 2 mM L-glutamine was prepared and 200 µl (≈20 000 cells) added to each well in 96-well tissue culture plates, excluding wells on outside edges of the plate which were filled with 200 µl phosphate buffered saline. Plates were incubated for 48 h at 37 °C in 5 % CO_2. 40 µl of culture medium was removed from each sample well and replaced with 40 µl of plasma sample. A positive (40 µl, 1 ng/ml Escherichia coli lipopolysaccharide) and negative control (40 µl of culture medium) was included in each plate. Each plasma sample and controls were run in duplicate. Plates were incubated at 37 °C in 5 % CO_2 for a further 24 h. SEAP activity was detected by addition of 20 µl supernatant to 180 µl alkaline phosphatase detection medium (QUANTi-Blue, InvivoGen, California, USA) in 96 well plates, which were incubated at 37 °C for 1 h. SEAP activity was assessed by reading the optical density (OD) at 620nm. Sample results were reported as response ratios relative to negative control i.e. mean sample OD divided by mean negative control OD.

IL-18/IL-1β reporter cell assay

Bioactive IL-18 and IL-1β in plasma samples was assayed using ‘HEK-Blue IL-18/IL-1 β’ cells (InvivoGen, California, USA), according to the manufacturer’s instructions. Binding of IL-18 and IL-1β to IL-18 receptor/ IL-18 receptor accessory protein and type 1 IL-1 receptor/Il-1 receptor accessory protein respectively activates NF-κB and AP-1 pathways resulting in SEAP expression. Assay details were as for TLR4 reporter assays with the following exceptions. Cells were seeded at an initial density of ≈50 000 cells/well and incubated for 24 h prior to addition of 20 µl plasma samples. Plasma from normal pigs after the initial surgical set up required for the porcine acute liver failure model were used as positive controls, as IL-18 has previously been shown to be elevated in acutely stressed pigs [3, 4]. SEAP activity was detected by addition of 40 µl supernatant to 160 µl alkaline phosphatase detection medium.

5. Liver histopathology results

Post mortem examination of liver specimens from pigs included in the main study to assess the efficacy of treatment with University College London-Liver Dialysis Device (UCL-LDD) compared to a Control Device (CD) in pigs with acetaminophen-induced acute liver failure (ALF) was performed. Acute centrilobular to midzonal hepatocyte degeneration and necrosis was confirmed in all ALF pigs irrespective of whether they were treated with UCL-LDD (APAP-UCL-LDD group) or CD (APAP-CD group), but not in any of the Control pigs (sham induction to ALF with water) treated with CD (Control-CD group) (Fig. 3). Necrosis was graded as mild, moderate or severe according to the degree and percentage of parenchyma affected. Median

grade for both APAP-UCL-LDD and APAP-CD groups was moderate with 4 out of 9 APAP-UCL-LDD pigs and 3 out of 7 APAP-CD having severe necrosis. There was no significant difference in severity of necrosis between APAP-UCL-LDD and APAP-CD.

6. Results for Il-18/Il-1β reporter cell assays

No significant IL-18/IL-1β bioactivity was detected in any of the plasma samples obtained after onset of acute liver failure from pigs treated with either University College London-Liver Dialysis Device (APAP-UCL-LDD group) or Control Device (APAP-CD group) when compared to normal control pigs treated with Control Device (Control-CD group) (Fig. 4).

Figures

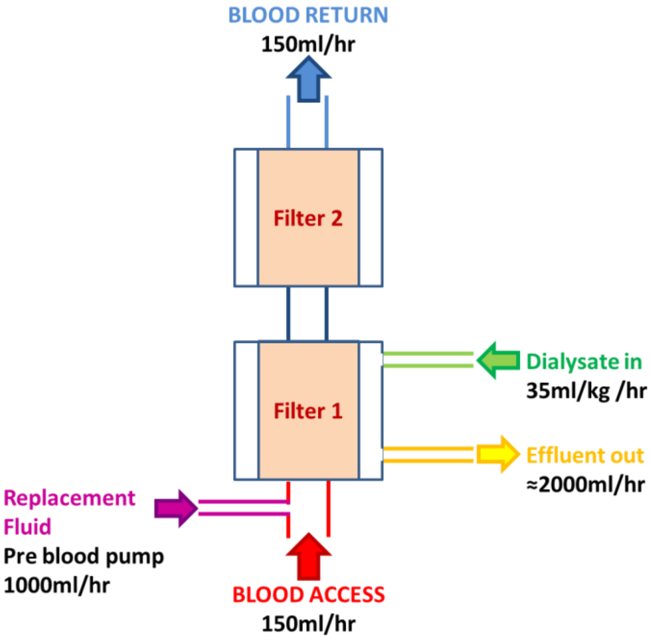

Fig. 1: Schematic representation of University College London-Liver Dialysis Device (UCL-LDD).

For UCL-LDD, Filter 1 was SepteX™ , a high cut-off filter and Filter 2 was OXiris™, an endotoxin absorption filter. UCL-LDD was compared to a Control Device (CD), in which Filter 1 and Filter 2 were standard continuous renal replacement filters, HF1000™. All components of the device were obtained from Gambro Dialysatoren GmbH, Rostock, Germany.

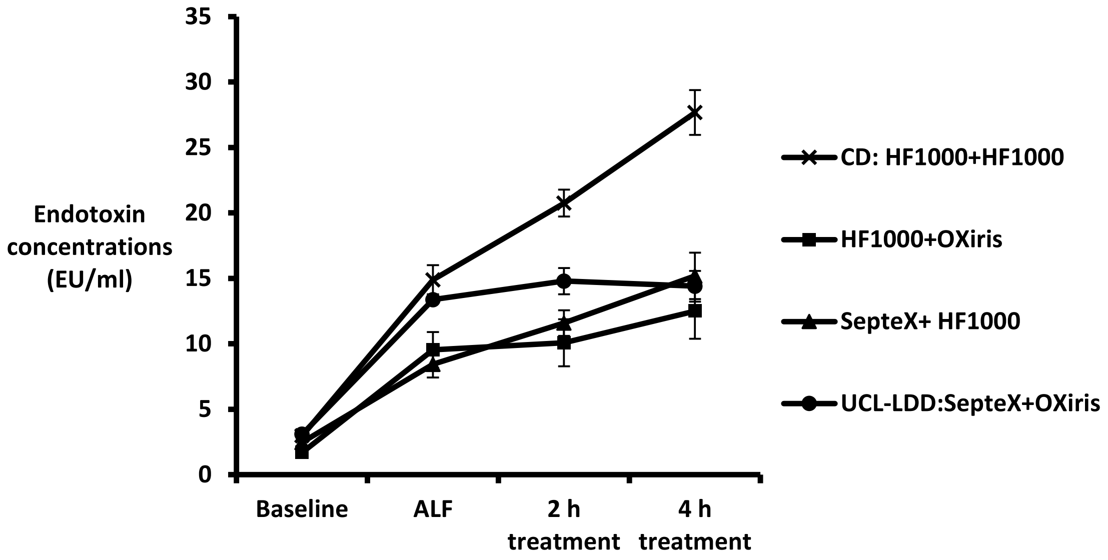

Fig. 2: Effect of treatment with four haemofilter combinations on endotoxin concentrations measured in plasma samples from pigs with acetaminophen-induced acute liver failure.

All plasma samples were diluted 1 in 10 and spiked with 5EU endotoxin. Endotoxin concentrations increased significantly in all groups from baseline to onset of acute liver failure (ALF). Thereafter further increase in endotoxin concentrations was inhibited by filter combinations including OXiris^{TM}, the endotoxin adsorption filter, but not by other filter combinations. (n = 3 per group)

(A.I)

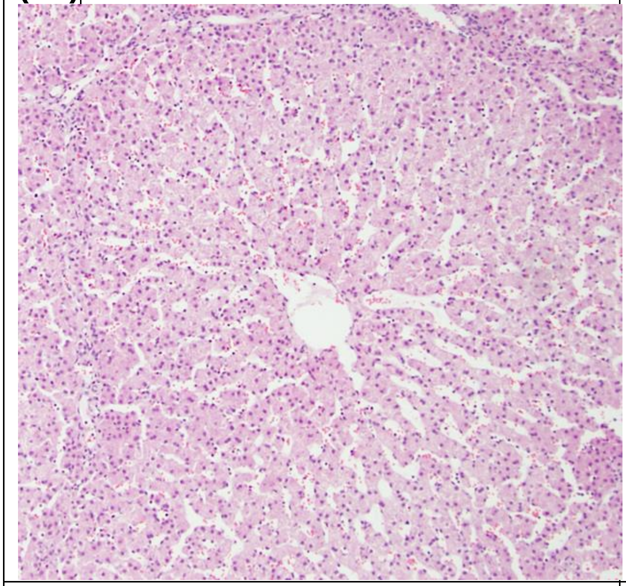

(A.II)

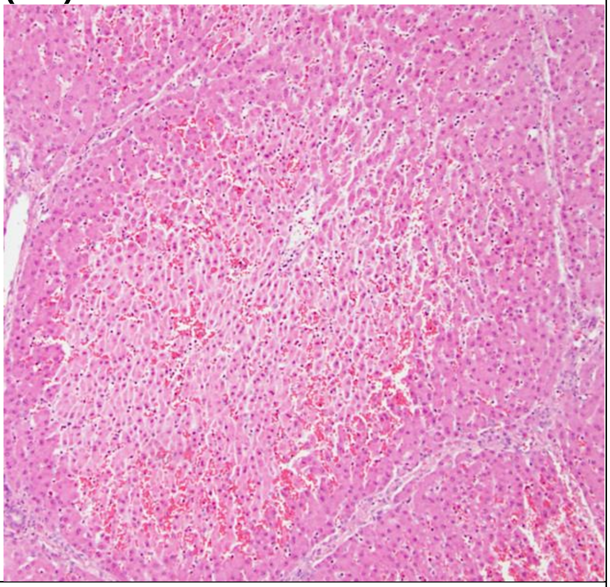

(B.I)

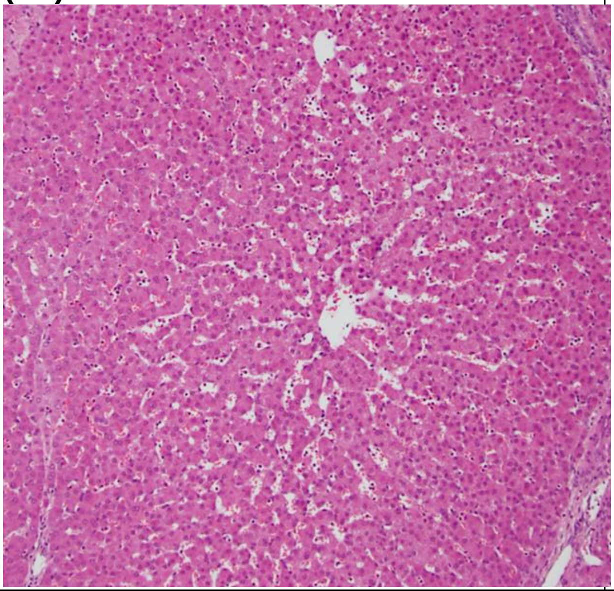

(B.II)

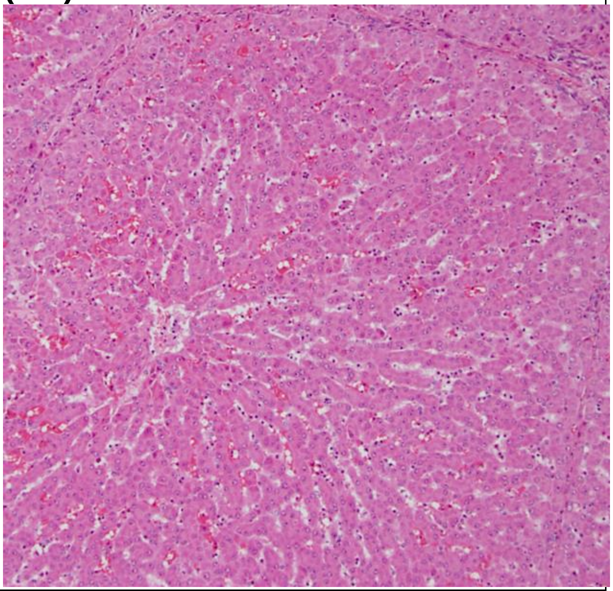

(C.I)

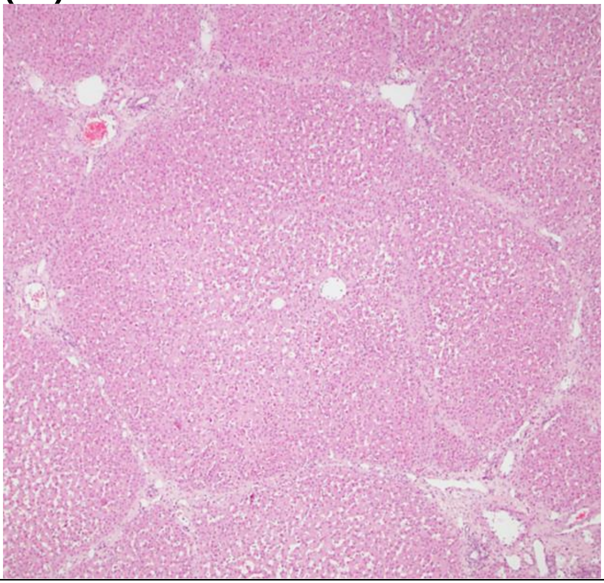

(C.II)

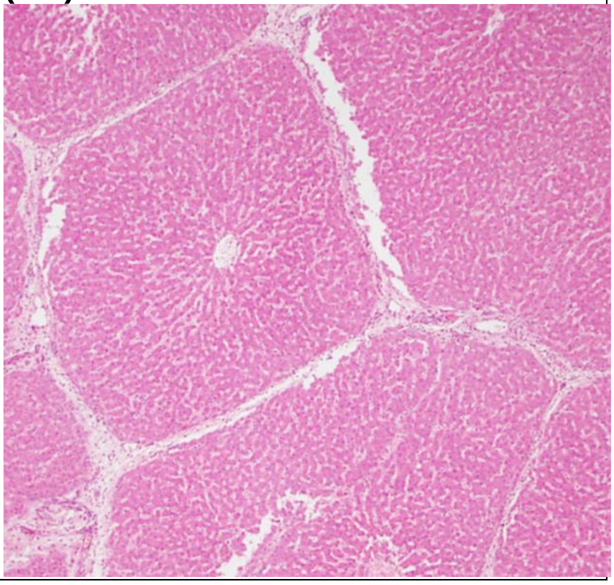

Fig. 3: Post-mortem examination of liver specimens from pigs included in the main study to assess the efficacy of treatment with University College London-Liver Dialysis Device (UCL-LDD) compared to a Control Device (CD) in pigs with acetaminophen-induced acute liver failure

Histopathology of pigs prior to acetaminophen dosing (I) and at post-mortem examination (II) in: (A) a pig with acute liver failure treated with CD with severe centrilobular to midzonal hepatocellular degeneration and necrosis with sinusoidal congestion and haemorrhage at post-mortem; (B) a pig with acute liver failure treated with UCL-LDD with multifocal (piecemeal), moderate centrilobular to midzonal hepatocellular degeneration and rare necrosis with mild sinusoidal congestion at post-mortem; and in (C) a normal control pig treated with CD with normal liver at post-mortem. (Haematoxylin and eosin stain, x40 magnification)

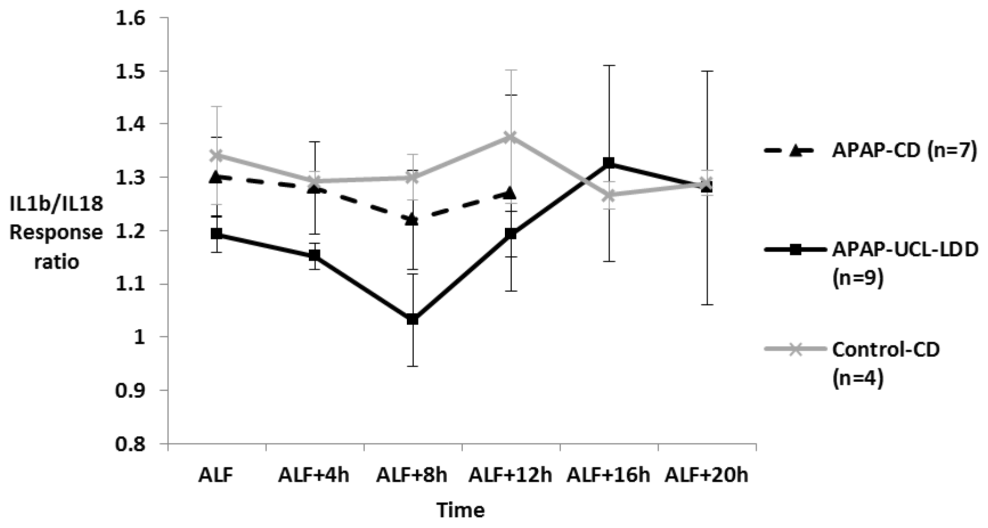

Fig. 4: IL-1b/IL-18 activity in plasma from acute liver failure pigs treated with either University College London-Liver Dialysis Device or Control Device

IL-1b/IL-18 activity was assessed using reporter cell assays. Response ratio (sample:negative control) for positive control was 1.958 ± 0.0898. Response ratios for acute liver failure pigs treated with either University College London-Liver Dialysis Device (APAP-UCL-LDD group) or Control Device (APAP-CD group) did not differ significantly from normal control pigs treated with Control Device (Control-CD group) at any time.

References

[1] Lee KC, Palacios Jimenez C, Alibhai H, Chang YM, Leckie PJ, Baker LA, et al. A reproducible, clinically relevant, intensively managed, pig model of acute liver failure for testing of therapies aimed to prolong survival. Liver Int 2013;33:544-551.

[2] Morgera S, Slowinski T, Melzer C, Sobottke V, Vargas-Hein O, Volk T, et al. Renal replacement therapy with high-cutoff hemofilters: Impact of convection and diffusion on cytokine clearances and protein status. Am J Kidney Dis 2004;43:444-453.

[3] Muneta Y, Minagawa Y, Nakane T, Shibahara T, Yoshikawa T, Omata Y. Interleukin-18 expression in pig salivary glands and salivary content changes during acute immobilization stress. Stress 2011;14:549-556.

[4] Barbe F, Atanasova K, Van Reeth K. Cytokines and acute phase proteins associated with acute swine influenza infection in pigs. Vet J 2011;187:48-53.
